# Impact of hepatocyte-specific deletion of staphylococcal nuclease and tudor domain containing 1 (SND1) on liver insulin resistance and acute liver failure of mice

**DOI:** 10.1080/21655979.2021.1974653

**Published:** 2021-10-05

**Authors:** Chunyan Zhao, Xiaoteng Cui, Yan Zhao, Baoxin Qian, Nan Zhang, Lingbiao Xin, Chuanbo Ha, Jie Yang, Xinting Wang, Xingjie Gao

**Affiliations:** a Department of Biochemistry and Molecular Biology, Department of Immunology, School of Basic Medical Sciences, Tianjin Medical University, Tianjin, China; b Key Laboratory of Immune Microenvironment and Disease, Ministry of Education, Key Laboratory of Cellular and Molecular Immunology in Tianjin, Excellent Talent Project, Tianjin Medical University, Tianjin, China; c Laboratory of Neuro-Oncology, Tianjin Neurological Institute, Department of Neurosurgery, Tianjin Medical University General Hospital and Key Laboratory of Neurotrauma, Variation, and Regeneration, Ministry of Education and Tianjin Municipal Government, Tianjin, China; d Department of Gastroenterology and Hepatology, The Third Central Clinical College of Tianjin Medical University, Tianjin Third Central Hospital, Tianjin, China

**Keywords:** SND1, insulin resistance, acute liver failure, conditional liver knockout, high-fat diet

## Abstract

Although our previous research shows an ameliorated high-fat diet (HFD)-induced hepatic steatosis and insulin resistance in global *SND1* transgenic mice, the involvement of SND1 loss-of-function in hepatic metabolism remains elusive. Herein, we aim to explore the potential impact of hepatocyte-specific *SND1* deletion on insulin-resistant mice. As SND1 is reported to be linked to inflammatory response, the pathobiological feature of acute liver failure (ALF) is also investigated. Hence, we construct the conditional liver knockout (LKO) mice of *SND1* for the first time. Under the condition of HFD, the absence of hepatic *SND1* affects the weight of white adipose tissue, but not the gross morphology, body weight, cholesterol level, liver weight, and hepatic steatosis of mice. Furthermore, we fail to observe significant differences in either HFD-induced insulin resistance or lipopolysaccharide/D-galactosamine-induced (LPS/D-GaIN) ALF between LKO and wild type (WT) mice in terms of inflammation and tissue damage. Compared with negative controls, there is no differential *SND1* expression in various species of sample with insulin resistance or ALF, based on several gene expression omnibus datasets, including GSE23343, GSE160646, GSE120243, GSE48794, GSE13271, GSE151268, GSE62026, GSE120652, and GSE38941. Enrichment result of SND1-binding partners or related genes indicates a sequence of issues related to RNA or lipid metabolism, but not glucose homeostasis or hepatic failure. Overall, hepatic SND1 is insufficient to alter the phenotypes of hepatic insulin resistance and acute liver failure in mice. The SND1 in various organs is likely to cooperate in regulating glucose homeostasis by affecting the expression of lipid metabolism-related RNA transcripts during stress.

## Graphical Abstract

**Figure:**
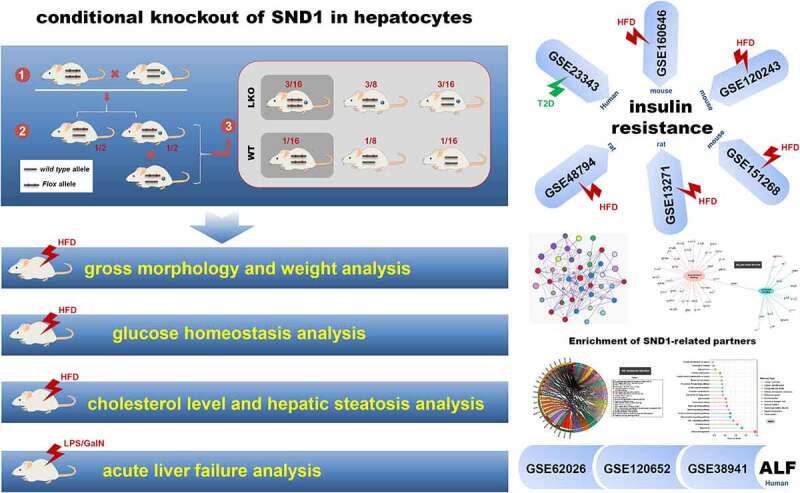

## 1. Introduction

Human SND1, also termed Tudor-SN (Tudor staphylococcal nuclease), contains four staphylococcal nucleases-like (SN1 ~ 4) domains at N-terminus and a Tudor-SN5 domain at C-terminus [1,2]. After a series of assays based on the cellular, animal, and clinical samples, *SND1* was reportedly involved in a variety of biological processes, such as gene transcription, splicing of mRNA precursors, cell cycle, DNA damage repair, proliferation, apoptosis, lipogenesis, and tumorigenesis [3–12].

Previously, we observed a reduced accumulation of triglyceride and the improved fatty liver and insulin resistance in the liver tissue of global *SND1* transgenic mice under the treatment of a high-fat diet [13]. As an essential organ of the body, the liver tissue is closely linked to the presence of insulin resistance [14]. Hepatocytes are responsible for lipogenesis, cholesterol biosynthesis, and glucose metabolism [15]. Growing evidence supports the functional links between SND1 protein and liver tissue in different species. For example, human SND1 can promote the proliferation of hepatocellular carcinoma cell lines [12,16] and takes part in the occurrence of hepatocellular carcinoma [17,18]. In the primary hepatocytes of rats, SND1 reportedly regulates the secretion of lipoprotein phospholipids [19,20]. Thus, we are interested in investigating the potential role of hepatic SND1 expression in the liver insulin resistance of mice.

Upon external stimuli, the liver organ may suffer from acute liver failure (ALF) [21]. ALF is a severe clinical syndrome that involves sudden and massive liver cell death and liver dysfunction, which may lead to the presence of coagulopathy, encephalopathy, and circulatory dysfunction [22,23]. It was reported that the highly expressed SND1 was associated with a chronic inflammatory state of hepatocellular carcinoma cells [18]. However, the relationship between SND1 expression and ALF with inflammation response remains elusive.

With regard to the *SND1* gene-modified animal models, only global *SND1* transgenic mice and hepatocyte-specific *SND1* transgenic mice (Alb/SND1 mice) [18] were studied previously [13]. In the present study, we constructed the liver *SND1* LKO mice using a Cre-loxP system for the first time. We then investigated whether hepatocyte-specific deletion of *SND1* in mice affects HFD-induced liver insulin resistance and LPS/D-GalN-induced ALF. Additionally, we performed the SND1 expression pattern analysis using the available gene expression omnibus (GEO) datasets for liver insulin resistance and acute liver failure and the enrichment analysis of SND1 binding or correlated partners.

## 2. Materials and methods

### 2.1. SND1 liver conditional knockout mice

We first constructed the *SND1 Flox*/*Flox* mice and produced the *SND1 Flox*/WT-albumin-Cre^+^ heterozygous mice by crossing the *SND1 Flox*/*Flox* mice and the albumin-Cre^+^ mice with the WT allele of *SND1* (WT/WT-albumin-Cre^+^mice, The Jackson Laboratory, USA). After the second round of mating, *SND1 Flox*/*Flox*-albumin-Cre^+^ homozygous mice were obtained as the *SND1* liver conditional knockout (LKO) mice.*SND1 Flox*/*Flox* littermates were used as WT controls.

To verify the successful construction of *SND1* LKO mice, we extracted the DNA from mouse primary hepatocytes and performed a genotyping polymerase chain reaction assay to detect the presence of *SND1 Flox* sites and the Cre gene. The primer sequences: *SND1 Flox* 5ʹ-CAGCACTAAAAGCTTGTCCC-3ʹ (Forward 1, F1), 5ʹ-ACGAGAGTATGGGATGATCT-3ʹ (Forward 2, F2), 5ʹ-GCTAAAGAGTCCCTAGAAAG-3ʹ (Reverse, R); *Cre* 5ʹ-GAAGCAGAAGCTTAGGAAGATGG-3ʹ (F), 5ʹ-TTGGCCCCTTACCATAACTG-3ʹ (R); Internal control 5ʹ-CAAATGTTGCTTGTCTGGTG-3ʹ (F); 5ʹ-GTCAGTCGAGTGCACAGTTT-3ʹ (R).

The mice were free to eat and drink under the feeding conditions [temperature of 22 ± 2°C, humidity: 40 ~ 70%; light cycle: 12/12 hours (h)]. A total of 10 *SND1* LKO male mice were randomly divided into two groups, namely the chow diet (10% kcal from fat, D12450B, Research Diets) (LKO CD) and high-fat diet group (60% kcal from fat, D12492, Research Diets) (LKO HFD). Meanwhile, the chow diet (WT CD) and high-fat diet (WT HFD) groups from the ten litters of WT male mice were included as controls. The body weight of WT or *SND1* LKO mice was measured every week. Mice were sacrificed by dislocation at the 24 weeks (w) of chow or high-fat diet. Liver and white adipose tissues were quickly separated and weighed. Each liver tissue was divided into three sections: 1) the first was used for the western blot analysis; 2) the second was for the detection of total liver cholesterol and liver-free cholesterol (Applygen, Beijing); 3) the third was subjected to Hematoxylin & Eosin (H&E) staining (ZSGB-BIO, China). The study protocols and use of animals were approved by the institutional animal care and use committee of Tianjin Medical University.

### 2.2. Primary hepatocyte extraction

We first anesthetized the mice by intraperitoneally injecting 7% chloral hydrate (25 mg/g). Five minutes later, the peritoneal cavity was opened. The inferior vena cava was perfused with EGTA solution (Sigma Aldrich). When the liver color became lighter, we cut the portal vein and perfused the liver using the solution of protease and collagenase (Sigma Aldrich). At the end of the perfusion, a white texture was visible on the liver. After perfusion, the mice were sacrificed, and the organ was cut and transferred to a 6 cm culture dish. After the addition of pre-warmed protease and collagenase *in vitro* hydrolyzate, we disrupted the liver tissue by forceps. Then, the broken liver tissue was transferred into a 50 ml centrifuge tube and incubated with the 20 ml pre-warmed protease and collagenase *in vitro* hydrolyzate and 1% DNase (Solarbio) in a 37°C hybridization chamber for 20 min. The liver tissue was then filtered by the 70 μm pore size Falcon filter (BD Biosciences Discovery Labware, Bedford, MA) and centrifuged 20 ~ 30 × g for 4 ~ 5 min at 4°C. The bottom primary hepatocytes were finally obtained.

### 2.3. Fasting/refeeding assay

After 4 w of chow or high-fat diet in mice, we performed a fasting/refeeding assay. Briefly, we fasted the mice for 16 h and measured the fasting blood glucose using a blood glucose meter (Accu-Chek Active, Roche). When the chow diet was restored, the blood glucose levels were measured at 0.5 h, 1 h, 2 h, 4 h, and 6 h, respectively. To assess the alteration of blood glucose, the area under the curve (AUC) was calculated as well.

### 2.4. Glucose and insulin tolerance test

As previously described [13], a glucose tolerance test was performed using the mice of CD (4 w), or HFD (4 w, 8 w, and 12 w). After the fasting treatment for 16 h, the mice were injected intraperitoneally with a glucose solution (1.5 g/kg). Additionally, an insulin tolerance test was performed [13] through the intraperitoneal injection of 0.75 U/kg insulin (solarbio) into the mice at the 16 w of chow or high-fat diet. We measured the blood glucose levels at the time points of 0 min, 15 min, 30 min, 45 min, 60 min, and 90 min, respectively, and calculated the AUC values.

### 2.5. Acute insulin response assay

As previously reported [13], we performed an acute insulin response assay. Briefly, at 24 w of chow or high-fat diet, WT, and *SND1* LKO mice were fasted overnight and anesthetized by intraperitoneal injection of 7% chloral hydrate (25 mg/g). Five minutes (min) later, we opened the abdominal cavity and injected the insulin solution (solarbio) into the portal vein. At the points of 0 and 5 min, tissue proteins in the liver leaves were extracted. The phosphorylation level of Akt protein was measured by a western blotting assay, using anti-Akt (Cell Signaling Technology) and anti-p-Akt (Cell Signaling Technology) antibodies. Then, the band density was digitized using an Image J 2X software (NIMH, Bethesda, MD, USA).

### 2.6. Western blot analysis

We performed a western blotting assay as previously described [6]. We homogenized the liver, spleen, pancreas, and kidney tissues of WT and LKO mice with a fast cell disrupter (Bullet Blender, Next advance) and isolated the primary hepatocytes. HCC SMMC-7721 cell line was provided by professor Zhi Yao (Tianjin Medical University). The following antibodies were used: anti-GAPDH (Proteintech Group), anti-β-actin (Sigma-Aldrich), anti-Akt (Cell Signaling Technology), and anti-p-Akt (Cell Signaling Technology). Mouse monoclonal anti-SND1 antibody was utilized as described previously [8,11].

### 2.7. ALF mice model

We established the mice model of ALF by intraperitoneally injecting LPS (5 mg/kg body weight; Sigma-Aldrich) and D-GalN (100 mg/kg body weight; Sigma-Aldrich) into *SND1* LKO or WT mice. Normal saline (NS) was used as a control. Serum aminotransferase activities of mice were measured using an aspartate aminotransferase (AST) detection kit (Jiancheng, Nanjing), an alanine aminotransferase (ALT) detection kit (Jiancheng, Nanjing), and a microplate reader (Varioskan Flash, Thermo). We also perform an H&E staining assay in different groups using Hematoxylin and eosin (ZSGB-BIO), according to the manufacturer’s instructions.

### 2.8. Quantitative real-time polymerase chain reaction (qPCR)

As described previously [12], we isolated the total RNA from the liver tissues of mice using TRIZOL reagent (Invitrogen) and synthesized the cDNA by a revert aid first-strand cDNA synthesis kit (Thermo Fisher Scientific). Then, FastStart universal SYBR Green Master Mix (Roche Diagnostics) was used to perform a polymerase chain reaction assay. The primer sequences: *IL-1β* [5ʹ-TGGACCTTCCAGGATGAGGACA-3ʹ (F); 5ʹ-GTTCATCTCGGAGCCTGTAGTG-3ʹ (R)]; *IL-6* [5ʹ-TACCACTTCACAAGTCGGAGGC-3ʹ (F); 5ʹ-CTGCAAGTGCATCATCGTTGTTC-3ʹ (R)]; *TNF-α* [5ʹ-GGTGCCTATGTCTCAGCCTCTT-3ʹ (F); 5ʹ-GCCATAGAACTGATGAGAGGGAG-3ʹ (R)].

### 2.9. GEO dataset analysis

As reported previously [24], we obtained the files of expression matrix, clinical trait, and platform annotation for the datasets of GSE23343, GSE160646, GSE120243, GSE48794, GSE13271, GSE151268, GSE62026, GSE120652, GSE38941, using an R package GeoQuery. Then, the extracted expression matrix of *SND1* in the liver or white adipose tissues was matched with the group information, such as control/type-2 diabetes (T2D), CD/HFD, control/ALF, using RStudio software version 1.3.1093. The result of GSE13271 was visualized as a curve plot using a GraphPad Prism software version 7.04. The other data was finally visualized as a bar plot or violin plot using the ggboxplot () or ggviolin () functions within R package ggpubr.

### 2.10. Enrichment analysis of SND1-related partners

Based on our previously reported datasets of SND1-binding partners [25] or GSE114447 [12], we performed the enrichment analysis of KEGG (Kyoto encyclopedia of genes and genomes) and GO (gene ontology), which includes the molecular function, cellular component, and biological process, using R package clusterProfiler [26,27]. The enrichment results of SND1-binding partners were visualized as a circleplot or classplot using a sangerbox tool (http://www.sangerbox.com/tool). For the GSE114447, we obtained the SND1-related differential genes in HepG2 cell lines using R package LIMMA [*P* < 0.05, fold change (FC)>1.2] and provided the volcano plot using R package ggplot2. The GO enrichment data was visualized as a cnetplot by the cnetplot () function, while KEGG data was visualized as a network plot by a metascape tool (https://metascape.org/).

### 2.11. Statistical analysis

Based on the IBM SPSS Statistics 19.0 software, we performed an independent Student’s t-test or one-way analysis of variance (ANOVA), followed by Tukey’s multiple comparison test. For the time-dependent datasets, we performed the two-way ANOVA with Sidak’s multiple comparison test using the GraphPad Prism software. Apart from the GSE13271 dataset, a wilcox.test was performed for other GEO datasets, using the compare_means () function of R package ggpubr. A *P* value of less than 0.05 was considered statistically significant.

## 3. Results

In this study, we first generated mice with the hepatocyte-specific deletion of *SND1* in the liver, namely *SND1* liver conditional knockout model. Then, we performed a series of experiments, such as fasting/refeeding assay, glucose/insulin tolerance test, and acute insulin response assay, to analyze the *in vivo* role of hepatic SND1 in the HFD-induced insulin resistance. We also investigated whether the hepatocyte-specific deletion of *SND1* influences the acute liver failure process by using an LPS/D-GalN-induced mice model of ALF. Further, we analyzed the SND1 expression pattern in various samples of GSE23343, GSE160646, GSE120243, GSE48794, GSE13271, GSE151268, GSE62026, GSE120652, and GSE38941, and the GO and KEGG enrichment analysis of SND1-binding or correlated partners,

### 3.1. Conditional knockout of SND1 in hepatocytes

**Figure 1.**
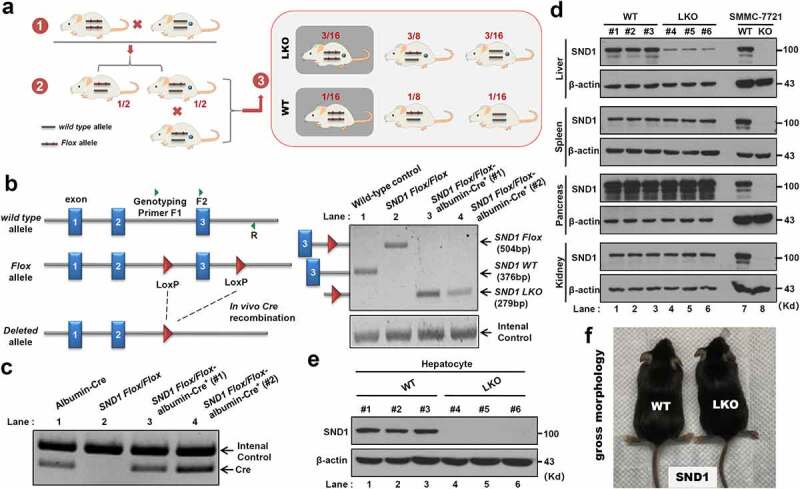
Construction of *SND1* LKO mice

To study the impact of hepatocyte-specific *SND1* deletion on liver insulin resistance and ALF, we first successfully constructed the liver LKO mice of *SND1*. As shown in Figure 1(a,b), we first generated the *SND1 Flox*/*Flox* mice with the *loxP* allele flanked at the exon 3 of the *SND1* gene and purchased the WT/WT-albumin-Cre^+^ mice, which contains the wild-type allele and the specific gene sequence for expressing albumin-induced Cre enzyme. By crossing the two strains mentioned above, the *SND1 Flox*/WT-albumin-Cre^+^ heterozygous mice were obtained from the offspring with a probability of 1/2. After another round of inbreeding *SND1 Flox*/WT-albumin-Cre^+^ heterozygous mice, the *SND1 Flox*/*Flox*-albumin-Cre^+^ homozygous mice (*SND1* LKO mice) were obtained with a probability of 3/16. *SND1 Flox*/*Flox* littermates were used as WT controls.

(a) The mating strategy for the construction of *SND1* LKO mice (b-c). We extracted the DNA from the primary hepatocytes of the wild-type (WT) control, *SND1 Flox/Flox, SND1 Flox/Flox*-albumin-Cre^+^ (#1, #2) mice, and performed a genotyping PCR assay. (d) We extracted the liver, spleen, pancreas, and kidney tissues from the WT (#1, #2, #3) and *SND1* LKO (#4, #5, #6) mice, respectively. SMMC-7721 cells of *SND1* WT and knockout (KO) were used as the controls. (e) We also extracted mouse primary hepatocytes. Western blotting was then performed using anti-SND1 or anti-β-actin antibodies. (f) The gross morphology of WT and LKO mice.

To determine the genotype of *SND1* LKO mice required for our experiments, we extracted the DNA from mouse primary hepatocytes and performed the genotyping assay using the mixture of three primers (Figure 1(b), F1 within intron 2 of *SND1*, F2 within exon 3, and R within intron 3) to detect the presence of *SND1* LoxP sites. For the WT control mice without LoxP site, *SND1* WT band of 376 bp was detectable through a PCR assay with the F2 plus R primers (Figure 1(b), lane 1). Due to the presence of the LoxP site within *SND1 Flox/Flox* mice, the *SND1 Flox* band of 504 bp was detectable using the primers of F2 plus R (Figure 1(b), lane 2). For the *SND1 Flox*/*Flox*-albumin-Cre^+^ homozygous mice (#1 and #2), the albumin-Cre contributed to *in vivo* Cre recombination between the two LoxP sites and the existence of *SND1* LKO band of 279 bp (Figure 1(b), lane 3 and 4, primers of F1 plus R). The internal control was detectable in all lanes (Figure 1b,c).

Besides, we extracted the liver, spleen, pancreas, and kidney tissues from the WT (#1, #2, #3) and *SND1* LKO (#4, #5, #6) mice, respectively (Figure 1(d)). SMMC-7721 cell lines of WT and *SND1* knockout (KO) were used as the controls. A western blotting assay was then performed using an anti-SND1 or anti-β-actin antibody. As shown in Figure 1(d), SND1 protein was utterly deleted in the SMMC-7721 of *SND1* KO. However, we only detected a diminished expression level of SND1 protein in the liver tissues of *SND1* LKO (#4, #5, #6) mice (Figure 1(d), lanes 1–3), compared with that of WT (#1, #2, #3) mice (lanes 4–6). There was no expression difference of SND1 between WT and *SND1* LKO *mice* in the tissues of spleen, pancreas, and kidney (Figure 1(d)). Further, we extracted primary hepatocytes from the liver tissue of the mice and observed that SND1 protein was deficient in the primary hepatocytes of *SND1* LKO mice (Figure 1(e)). The above indicated a workable system of mice with the hepatocyte-specific deletion of *SND1 was* successfully constructed. We also provided the gross morphology of WT and *SND1* LKO mice in Figure 1(f) and did not observe a significant difference.

### 3.2. Gross morphology and weight analysis

**Figure 2.**
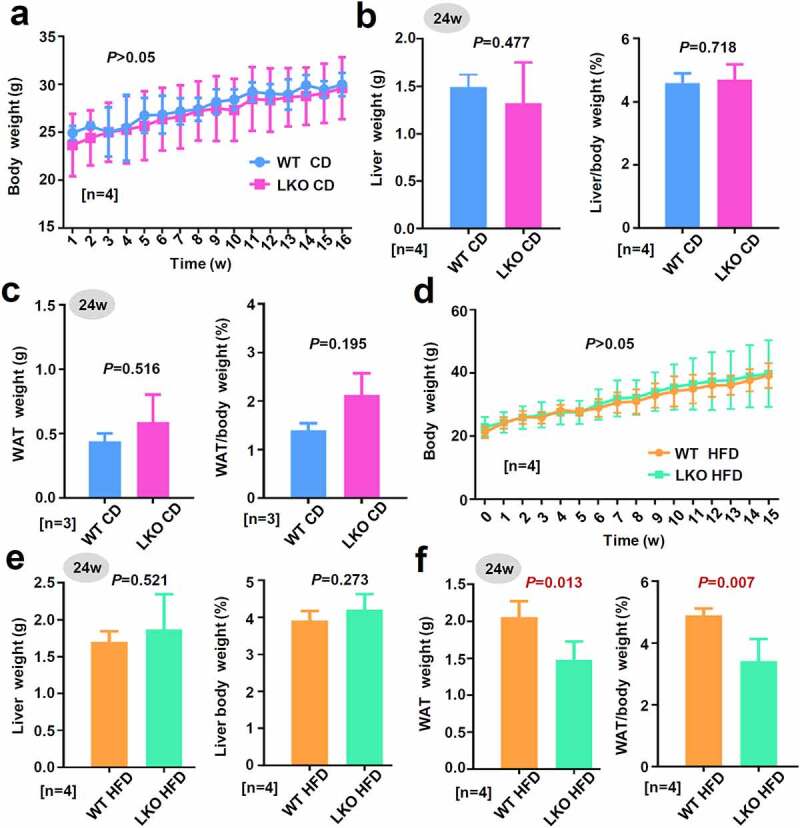
Effect of *SND1* hepatocyte-specific deletion on weight of mice

We then investigated the effect of *SND1* liver conditional knockout on the weight of mice on a chow diet (CD) or high-fat diet (HFD). For the CD group, we monitored the body weights of WT or *SND1* LKO mice weekly and did not detect a significant change (Figure 2(a), *P* > 0.05) between WT and LKO over different time points. After 24 w of CD, we measured the weight of liver tissue and calculated the ratio value of liver/body weight. There were no statistical deviations between the WT and *SND1* LKO mice (Figure 2(b), *P* = 0.477 for liver weight, *P* = 0.718 for the liver weight/body weight). We further measured the weight of white adipose tissue (WAT) and calculated the ratio value of WAT/body weight, respectively, neither of which showed a significant difference as shown in Figure 2(c) (*P* = 0.516 for WAT weight, *P* = 0.195 for the WAT/body weight).

(a) The body weight of WT or LKO mice with chow diet (CD) was measured every week, respectively. Then, the statistical analysis of two-way ANOVA with Sidak’s multiple comparisons was performed. The *P* values of Sidak’s multiple comparisons at the different time points were indicated. (b-c) At the 24 w of CD in mice, the weights of extracted liver tissue and white adipose tissue (WAT) were measured, respectively. And the ratio value of liver/body weight, or WAT/body weight, was calculated. Student’s t-test was then performed. (d-f) A similar measure was performed for the mice with a high-fat diet (HFD).

**Figure 3.**
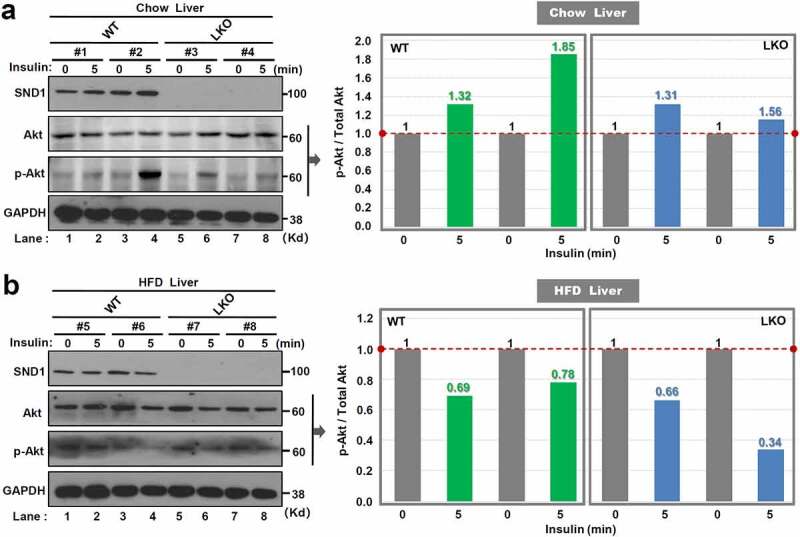
Effect of *SND1* hepatocyte-specific deletion on Akt pathway

At the 24 w of chow diet in mice, *SND1* WT (a) and LKO (b) mice were fasted overnight and anesthetized. After the injection of the insulin solution, the phosphorylation level of Akt protein in the liver tissue was analyzed by a western blotting assay at the time points of 0 min and 5 min. The band density was digitized by the Image J 2X software. The value of p-Akt/Total Akt was indicated.

**Figure 4.**
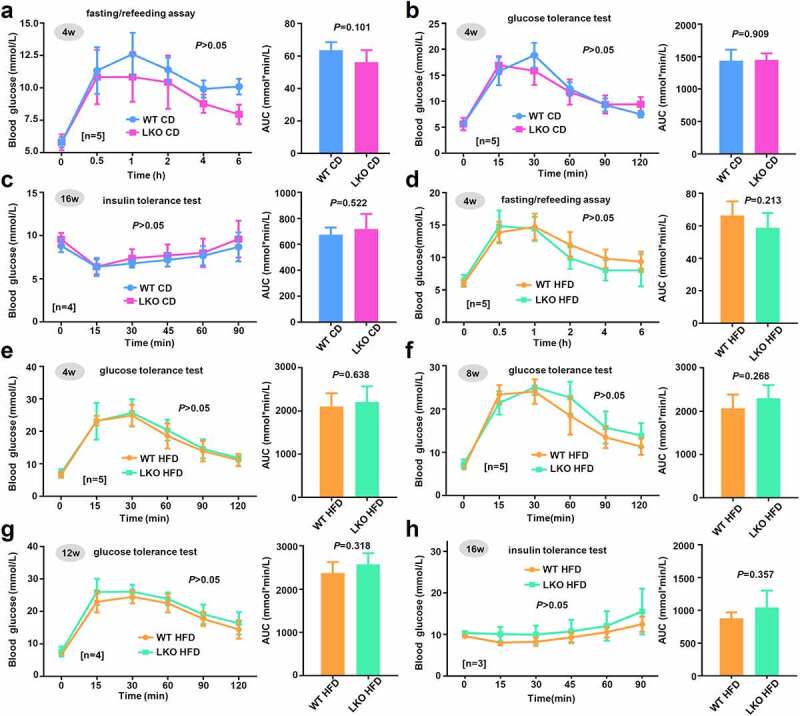
Effect of *SND1* hepatocyte-specific deletion on glucose homeostasis of mice

(a) After the fasting treatment of mice with 4 w CD for 16 h, the chow diet was restored. The blood glucose levels at the time points of 0 h, 0.5 h, 1 h, 2 h, 4 h, and 6 h were measured, respectively. (b) After the fasting treatment for 16 h, 1.5 g/kg glucose solution was injected intraperitoneally into WT or *SND1* LKO mice with 4 w chow diet. The blood glucose levels at the time points of 0 min, 15 min, 30 min, 60 min, 90 min, and 120 min were measured, respectively. (c) 0.75 U/kg insulin was injected intraperitoneally into WT or *SND1* LKO mice with 16 w chow diet. The blood glucose levels at the time points of 0 min, 15 min, 30 min, 45 min, 60 min, and 90 min were measured. (d-f) The similar measure was performed for the mice with HFD. Two-way ANOVA with Sidak’s multiple comparison test was performed. In addition, the AUC value of the above was calculated, respectively. Statistical difference was analyzed by a Student’s t-test.

**Figure 5.**
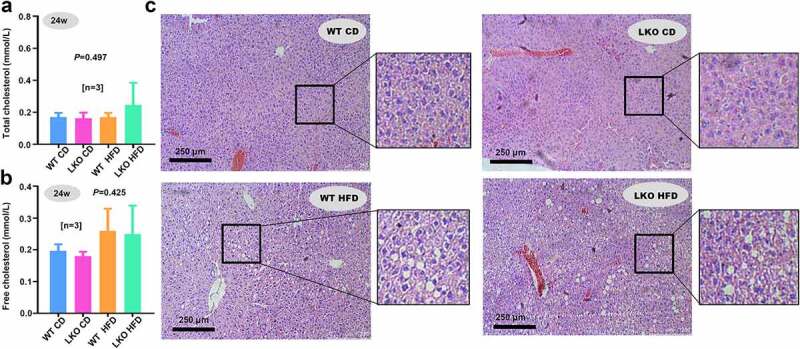
Cholesterol level and hepatic steatosis in WT and LKO mice with a high-fat diet

(a-c) At 24 w of a chow or high-fat diet, the levels of liver total cholesterol and liver-free cholesterol were measured, respectively. The ANOVA followed by Tukey’s multiple comparison test was performed for the statistical difference among different groups. (d) Liver sections were subjected to H&E staining, and images were captured by an optical microscope. Bar scale, 250 μm.

**Figure 6.**
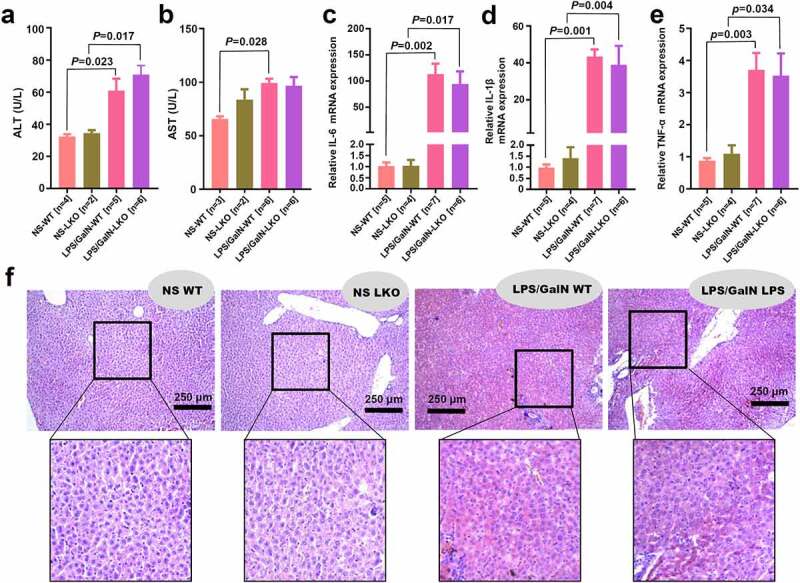
Effect of *SND1* hepatocyte-specific deletion on acute liver failure induced by LPS/D-GalN

WT and LKO mice were administered 5 mg/kg LPS and 100 mg/kg D-GalN intraperitoneally. Normal saline (NS) was used as the control. Serum activities of ALT (a) and AST (b) were measured after LPS/D-GalN treatment for 6 h. The mRNA levels of *IL-6* (c), *IL-1β* (d), and *TNF-α* (e) in the liver tissues were also measured by a qPCR assay. The ANOVA followed by Tukey’s multiple comparison test was performed. (f) H&E staining images of representative liver samples were provided. Scale bar, 250 μm.

**Figure 7.**
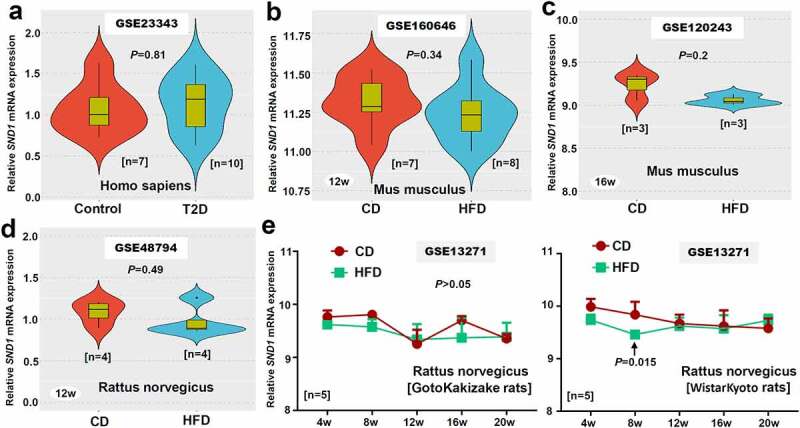
*SND1* expression in liver tissues of insulin resistance-related GEO datasets

(a) For GSE23343, the expression difference of human *SND1* mRNA in the liver tissues between the T2D patients with insulin resistance (n = 7) and controls (n = 10) was analyzed using a wilcox.test. (b-d) We also analyzed the statistical difference of *SND1* expression in liver tissue between the CD and HFD of mice (Mus musculus) or rats (Rattus norvegicus). (e) For GSE13271, we performed the two-way ANOVA with Sidak’s multiple comparison test to analyze the expression difference of *SND1* in liver tissues of GotoKakizake or WistarKyoto rats between CD and HFD at the time points of 4 w, 8 w, 12 w, 16 w, 20 w. The positive *P* value was indicated.

**Figure 8.**
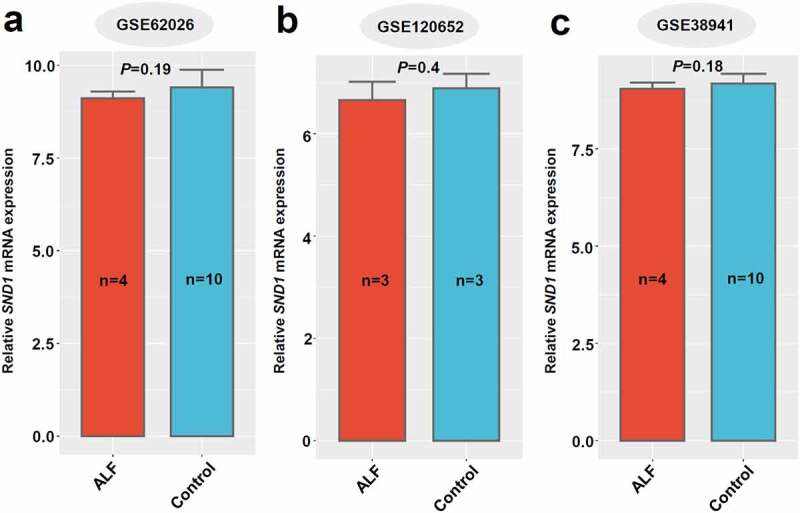
*SND1* expression in liver tissues of ALF-related GEO datasets

We performed a wilcox.test to analyze the statistical difference of *SND1* expression in the liver tissues between ALF and control, based on the datasets of GSE62026 (a), GSE120652 (b), and GSE38941 (c).

For the HFD group, we also failed to observe the significant difference in body weight (Figure 2(d), *P* > 0.05; Figure 2(e), *P* = 0.521) and the ratio value of liver/body weight (Figure 2(e), *P* = 0.273) between WT HFD and LKO HFD mice. Nevertheless, the absence of hepatic *SND1* resulted in a decreased WAT weight (Figure 2(f), *P* = 0.013) or the ratio value of WAT/body weight (*P* = 0.007). These data indicated that *SND1* liver conditional deficiency decreased the weight of white adipose tissue in mice under the condition of a high-fat diet, but not the gross morphology, body weight, and liver weight of mice.

### 3.3. Glucose homeostasis analysis

Considering the links of insulin stimulation to Akt pathway activation [13,28], we performed an acute insulin response assay to analyze the effect of *SND1* hepatocyte-specific deletion on phosphorylation modification of Akt protein. After the 24 w of chow or high-fat diet, *SND1* WT, and LKO mice were fasted overnight and anesthetized. After the injection of the insulin, the phosphorylation level of Akt protein in the liver tissue was analyzed by western blotting assay at the point of 0 min and 5 min. As shown in Figure 3(a), insulin treatment can increase the phosphorylation level of Akt protein in the liver tissue of WT mice. However, we observed a downward trend of the Akt phosphorylation signal in LKO mice (Figure 3(a)). In addition, there was a decreased phosphorylation level of Akt protein in both WT and LKO mice with 24 w of HFD (Figure 3(b)), suggesting that high-fat diet results in the occurrence of insulin resistance. Compared with WT mice, we observed a lower level of Akt protein phosphorylation in LKO mice with HFD (Figure 3(b)). These results indicate a marginally suppressive effect of *SND1* hepatocyte-specific deletion on the activation of Akt pathway during insulin stimulation.

Next, we determined to analyze the glucose homeostasis of WT and *SND1* KO mice on a chow or high-fat diet. A fasting/refeeding assay was carried out. After the fasting treatment for 16 h, we restored the chow diet and measured the blood glucose levels of mice on 4 w CD at 0 h, 0.5 h, 1 h, 2 h, 4 h, and 6 h, respectively. As shown in Figure 4(a), there was no statistical difference in blood glucose alteration between WT CD and *SND1* LKO CD mice (*P* > 0.05 for all time points; *P* = 0.101 for AUC). Then, a glucose tolerance test was performed. After the fasting treatment for 16 h, we injected intraperitoneally 1.5 g/kg glucose solution into the WT or *SND1* LKO mice on 4 w CD and measured the blood glucose levels at the time points of 0 min, 15 min, 30 min, 60 min, 90 min, and 120 min, respectively. As shown in Figure 4(b), similar negative results were obtained (*P* > 0.05 for all time points; *P* = 0.909 for AUC). Further, we performed an insulin tolerance test at the 16 w of chow in mice. The 0.75 U/kg insulin was injected intraperitoneally into the mice, and the blood glucose levels at the time points of 0 min, 15 min, 30 min, 45 min, 60 min, and 90 min were measured. As shown in Figure 4(c), compared with the WT CD, no increased blood glucose level (*P* > 0.05) or AUC (*P* = 0.522) was observed in the *SND1* LKO CD. Moreover, we performed a fasting/refeeding assay (Figure 4(d)), glucose tolerance test (Figure 4(e–g)), and insulin tolerance test (Figure 4(h)), respectively, in the WT and *SND1* LKO mice on a high-fat diet. Also, we did not observe the statistical difference between the WT and *SND1* LKO mice (Figure 4(d–h), all *P* > 0.05). Taken together, *SND1* liver conditional deficiency is unable to modulate the glucose homeostasis of mice under the condition of either a chow diet or a high-fat diet.

### 3.4. Cholesterol level and hepatic steatosis analysis

Previously, we reported that SND1 could regulate the cholesterol metabolism of mice by promoting the activity of sterol regulatory element-binding protein 2 under the condition of HFD [13]. Also, the highly expressed SND1 expression affects the cholesterol distribution and homeostasis of hepatocellular carcinoma cells [29]. To this end, we sought to detect the cholesterol level of both WT and *SND1* LKO mice. Unexpectedly, we did not observe the statistical difference of the total cholesterol (Figure 5(a), *P* = 0.497) or free cholesterol (Figure 5(b), *P* = 0.425) level between WT and *SND1* LKO mice on either chow or high-fat diet. Furthermore, the H&E staining data of liver sections (Figure 5(c)) indicated a significant increase in fat vacuoles of HFD mice compared with that of CD mice. However, we did not observe a significant difference between WT and *SND1* LKO mice. This suggested that hepatocyte-specific deletion of *SND1* is unlikely to modulate the cholesterol level and hepatic steatosis of mice.

### 3.5. Hepatic failure analysis

It has been reported that SND1 is related to the inflammation response [18]. We thus sought to investigate the potential effect of *SND1* hepatocyte-specific deletion on the LPS/D-GalN-induced acute liver failure of mice. The serum levels of ALT or AST were measured. Compared with the NS control group, we observed an increased level of ALT in both WT (Figure 6(a), *P* = 0.023) and *SND1* LKO (*P* = 0.017) mice under 6 h of LPS/D-GalN stimulation. Nevertheless, there was no statistical difference between WT and *SND1* LKO mice (Figure 6(a)). Moreover, we observed that LPS/D-GalN stimulation led to an increase AST level in the WT mice (Figure 6(b), *P* = 0.028), but not LKO mice (Figure 6(b), *P* > 0.05). There was also no statistical difference of AST between WT and LKO mice (Figure 6(b)).

The quantitative RT-PCR assay was conducted to measure the levels of three inflammatory cytokines, including *IL-6, IL-1β*, and *TNF-α*. As shown in Figure 6(c), the LPS/D-GalN treatment could induce an increased level of *IL-6* expression in both WT (*P* = 0.002) and *SND1* LKO (*P* = 0.017) mice. Similar results were obtained for the *IL-1β* (Figure 6(d), *P* = 0.001 for WT; *P* = 0.004 for LKO) and *TNF-α* (Figure 6(e), *P* = 0.003 for WT; *P* = 0.034 for LKO). Consistently, there was no statistical difference of *IL-6, IL-1β*, and *TNF-α* in the presence and absence of hepatic *SND1* (Figure 6(c–e)). We further performed an H&E staining assay to assess the effect of LPS/D-GalN on the histomorphology of liver tissue and explored the potential influence of hepatocyte-specific deletion of *SND1* on the LPS/D-GalN-induced liver damage. As shown in Figure 6(f), LPS/D-GalN treatment led to significant liver damage (e.g., liver tissue hemorrhage and inflammatory cell infiltration) in *SND1* LKO mice, compared with the WT mice. Nevertheless, we failed to observe the statistically significant differences regarding the extent of liver damage between WT and *SND1* LKO mice. Taken together, the current results suggest that hepatocyte *SND1* is unable to contribute to altering hepatic inflammatory tones and tissue damage in acute liver failure of mice.

### 3.6. Further analysis of external GEO datasets

In addition to the above *in vivo* animal experiments that showed SND1 loss of function in the liver is incapable of changing the phenotypes in either insulin resistance or acute liver failure of mice, we sought external validation on various species of samples by utilizing the accessible datasets in terms of HFD-induced insulin resistance or ALF from GEO or Array Express database. After database retrieval, we got access to six insulin resistance-related human datasets, including GSE23343, GSE160646, GSE120243, GSE48794, GSE13271, and GSE151268, among which is GSE23343 that contains the expression data of *SND1* in the liver tissues of seven T2D patients with insulin resistance and ten negative controls. Thus, we analyzed the expression feature of *SND1* and failed to observe the statistical difference (Figure 7(a), *P* = 0.81). For other GEO datasets, the *SND1* expression level in the liver or white adipose tissues of HFD-induced mice or rat models was screened out. Still, we observed a similar negative correlation in mouse or rat samples (Figure 7(b–e) and Figure S1a-d, *P* > 0.05), albeit the liver tissues of WistarKyoto rats at 8 w (Figure 7(e), *P* = 0.015) and the white adipose tissues of GotoKakizake rats at 12 w (Figure S1c, *P* = 0.002).

In addition, we obtained three ALF-related datasets (GSE62026, GSE120652, and GSE38941) that contain the *SND1* expression data in the liver tissue of clinical patients with ALF. As shown in Figure 8, there was no statistical significance between ALF and control, even though we detected a decreased trend in the liver tissues of patients with ALF, compared with controls (all *P* > 0.05). Overall, the hepatic expression of *SND1* in pathological models of insulin resistance and acute liver failure remains stable, indicating a dispensable role of endogenous SND1 in nonmalignant hepatocytes.

### 3.7. Enrichment analysis of SND1-related partners

Previously, we performed a microarray assay using the HepG2 stable cell lines with the knockdown of *SND1* [12], from which the related chip data was accessed in the GEO database with the accession number ‘GSE114447’ [12]. As shown in Figure S2a, a total of 2,180 up-regulated and 1,228 down-regulated mRNA or lncRNA were identified. Our KEGG enrichment data (Figure S2b) showed the association between the SND1-related genes and the pathways of ‘fatty acid metabolism’/‘cholesterol biosynthesis’/‘glycerolipid metabolism’. Further, the ‘phospholipid binding’ issue was enriched in the ‘GO_molecullar function’ analysis (Figure S3). We did not observe the significant enrichment of glucose homeostasis or ALF-related issues. Besides, we performed the GO and KEGG enrichment analyses of SND1-binding partner [25] and identified a series of RNA metabolism-related issues, but not the pathways related to glucose homeostasis or hepatic failure (Figure S4-S7). Given the close links or multicellular synergism between glucose and lipid metabolism [30], SND1 in different organs is likely to cooperate in regulating glucose homeostasis by altering the expression of a set of lipid metabolism-related RNA transcripts under specific stress conditions.

## 4. Discussion

Although we have previously implicated gain of function of SND1 in improving insulin resistance in the global *SND1* transgenic mice in an HFD-induced mouse model [13], the loss of function of SND1 in the liver in regulating pathophysiologic function is unclear. In this study, we successfully constructed *SND1* liver-specific knockout mice for the first time and investigated the potential role of endogenous SND1 in an HFD-induced liver insulin resistance model and acute liver failure model. We failed to observe the significant difference in phenotypes from either liver insulin resistance or acute liver failure between WT and *SND1* LKO mice, even though there is somewhat an improved effect of *SND1* on the activation of the Akt pathway upon insulin stimulation in the liver from HFD-mice. Based on the GEO datasets, there was no differential *SND1* expression between normal and pathologic samples, suggesting a dispensable role of endogenous SND1 in nonmalignant hepatocytes. This is in accordance with our previous study in which we have shown that the improved cholesterol homeostasis in HFD-fed transgenic mice is attributable to the less repressive impact on the cholesterol pathway caused by HFD instead of an inductive influence [13]. The hepatic and global insulin sensitivity derived from HFD-transgenic mice is likely to dominate cholesterol homeostasis rather than endogenously hepatic SND1 itself, which also rendered us speculate the dispensable role of SND1 from normal hepatocytes in regulating cholesterols. Therefore, it appears that the synergistic action of SND1 in multiple organs contributes to the alteration of the metabolic phenotype.

In this study, we observed the decreased weight of white adipose tissue in the *SND1* liver conditional knockout mice under the condition of a high-fat diet, but not the gross morphology, body weight, and liver weight. Although the hepatocyte-specific deletion of *SND1* does not remarkably influence liver insulin resistance, it is clearly warranted to analyze further the potential effects of *SND1* in adipose or muscle tissues. Some studies hint at the possible link between cholesterol metabolism and SND1 expression. Based on the global *SND1* transgenic mice, we previously identified the promotive role of SND1 in cholesterol homeostasis, solely under the treatment of a high-fat diet but not the chow diet [13]. In line with our previous data, this study shows no alteration of cholesterols when SND1 is deficient in hepatocytes. Intriguingly, some reflections on malignant hepatic cells have shown the opposite. In hepatocellular carcinoma, the up-regulation of SND1 expression can influence the cellular cholesterol distribution and homeostasis [29]. The overexpression of SND1 protein in hepatoma cells contributes to the accumulation of cholesteryl esters, leading to more cholesterol esterification via fatty acid and the limitation of triglyceride synthesis [31]. This phenomenon was not observed here in the *SND1* liver-specific knockout mice. How to explain this? We believe that metabolic reprogramming is essential for maintaining the growth and proliferation of cancer cell [32], which may partly explain the difference between hepatoma cells and mice normal hepatocytes.

LPS from gram-negative bacteria is implicated in the pathogenesis of ALF [33]. LPS interacts with CD14 and Toll-like receptor 4 to activate inflammatory signals and induce the secretion of pro-inflammatory cytokines, which stimulates the infiltration of inflammatory cells into the liver [34–36]. SND1 is reported to be associated with activation of NF-κB that involves a chronic inflammatory state leading to HCC [18,37–39]. Here, we aimed at further investigating whether the hepatocyte-specific deletion of *SND1* in mice affects the presence of acute liver failure induced by LPS/D-GalN. Our results did not show the remarkable difference for the LPS/D-GalN-induced secretion of the pro-inflammatory cytokines (IL-1β, IL-6, and TNF-α) and acute liver failure histomorphology between WT and *SND1* LKO mice. Upon stimulation by LPS, the liver resident macrophages, named Kupffer cells, mainly initiate the inflammatory response by secreting pro-inflammatory cytokines, such as IL-1β, IL-6, and TNF-α [40,41]. Although hepatocyte-specific deletion of *SND1* does not remarkably influence the presence of acute liver failure, it is still worth a study to further analyze the potential effects of SND1 in the global deletion mice.

Our findings demonstrate a dispensable role of hepatic endogenously SND1 in insulin resistance and acute liver failure. However, no more than 10 mice with *SND1* knocked out were finally enrolled for our analyses due to our limited experimental condition. The variation across the mice weakens the effectiveness of statistical analyses to some extent. Thus, we try to remove the possible effect of a single outlier and comprehensively assess the influence of hepatocyte-specific *SND1* deletion in the glucose homeostasis through a set of assays, such as fasting/refeeding assay, glucose and insulin tolerance, and acute insulin response. Additionally, we analyzed the potential correlations of *SND1* expression with insulin resistance and acute liver failure in the species of humans, mice, and rats, based on a set of GEO datasets. The potential pathway enrichment analyses of SND1 interacting or related partners were performed as well, respectively. The negative but reliable conclusions were considered in this work. However, the synergistic mechanism of multiple organs regarding the role of SND1 in insulin sensitivity and inflammatory response still merits further experiments.

## 5. Conclusion

In summary, we, for the first time, successfully established a mouse model of *SND1* LKO, which serves as a valuable tool for further investigating the role of hepatic *SND1*. Our *in vivo* experimental results suggest a dispensable role of endogenously hepatic SND1 in the HFD-induced insulin resistance or LPS/D-GalN-induced acute liver failure in mice. The SND1 synergistic action from multiple organs may contribute to the liver insulin sensitivity or inflammatory response during stress.

## Supplementary Material

Supplemental Material

## Data Availability

The datasets used and/or analyzed during the current study are available from the corresponding author on reasonable request. The authors gratefully acknowledge the contributions of datasets within GEO database, including GSE23343, GSE160646, GSE120243, GSE48794, GSE13271, GSE151268, GSE62026, GSE120652, GSE38941.

## Highlight

- The first successful construction of liver *SND1* conditional knockout mice.
- Hepatocyte-specific *SND1* deletion cannot affect HFD-induced liver insulin resistance.
- There is no difference in LPS/D-GalN-induced ALF between LKO and WT mice.
- SND1-binding partners or related genes are related to RNA or lipid metabolism.
- SND1 of multiple organs may synergistically regulate insulin sensitivity.
