# Supplementary material for: Impact of hepatocyte-specific deletion of staphylococcal nuclease and tudor domain containing 1 (SND1) on liver insulin resistance and acute liver failure of mice
Source: Bioengineered. 2021 Oct 5;12(1):7360–75. doi: 10.1080/21655979.2021.1974653 (PMC8806720; doi:10.1080/21655979.2021.1974653)
Supplement: Supplemental Material [file KBIE_A_1974653_SM9149.zip › supplementary/Supplemental data.docx]

**Supplemental data**

**Figure S1. *SND1* expression in** **white adipose tissues of insulin resistance-related GEO datasets.**

We analyzed the statistical difference of *SND1* expression in white adipose tissues between CD and HFD based on the datasets of GSE151268 (a) and GSE120243 (b). For GSE13271, Two-way ANOVA with Sidak’s multiple comparisons test was performed to analyze the expression feature of *SND1* in white adipose tissues of GotoKakizake (c) or WistarKyoto (d) rats at the time points. The positive *P* value was indicated.

**Figure S2.** **KEGG enrichment analysis of SND1-related genes.**

Based on the dataset of GSE114447, the SND1-related differential genes in HepG2 cell lines were obtained and visualized as a volcano plot (a). Then, the KEGG enrichment data was visualized as a network plot (b).

**Figure S3.** **Molecular function enrichment analysis of SND1-related genes.**

Based on the dataset of GSE114447, the GO_molecular function enrichment analysis of SND1-related genes was performed using R package clusterProfiler. The data was visualized as a cnetplot.

**Figure S4. Molecular function enrichment analysis of SND1-binding partners.**

Based on the R package clusterProfiler, the GO_molecular function enrichment analysis of SND1-binding partners was performed. The data was visualized as a Circleplot.

**Figure S5. Cellular component enrichment analysis of SND1-binding partners.**

Based on the R package clusterProfiler, the GO_cellular component enrichment analysis of SND1-binding partners was performed. The data was visualized as a Circleplot.

**Figure S6. Biological process enrichment analysis of SND1-binding partners.**

Based on the R package clusterProfiler, the GO_biological process enrichment analysis of SND1-binding partners was performed. The data was visualized as a Circleplot.

**Figure S7. KEGG enrichment analysis of SND1-binding partners.**

Based on the R package clusterProfiler, the KEGG enrichment analysis of SND1-binding partners was performed. The data was visualized as a Classplot.
