# Supplementary material for: Impact of hepatocyte-specific deletion of staphylococcal nuclease and tudor domain containing 1 (SND1) on liver insulin resistance and acute liver failure of mice
Source: Bioengineered. 2021 Oct 5;12(1):7360–75. doi: 10.1080/21655979.2021.1974653 (PMC8806720; doi:10.1080/21655979.2021.1974653)
Supplement: Supplemental Material [file KBIE_A_1974653_SM9149.zip › supplementary/Supplementary Figure.pdf]

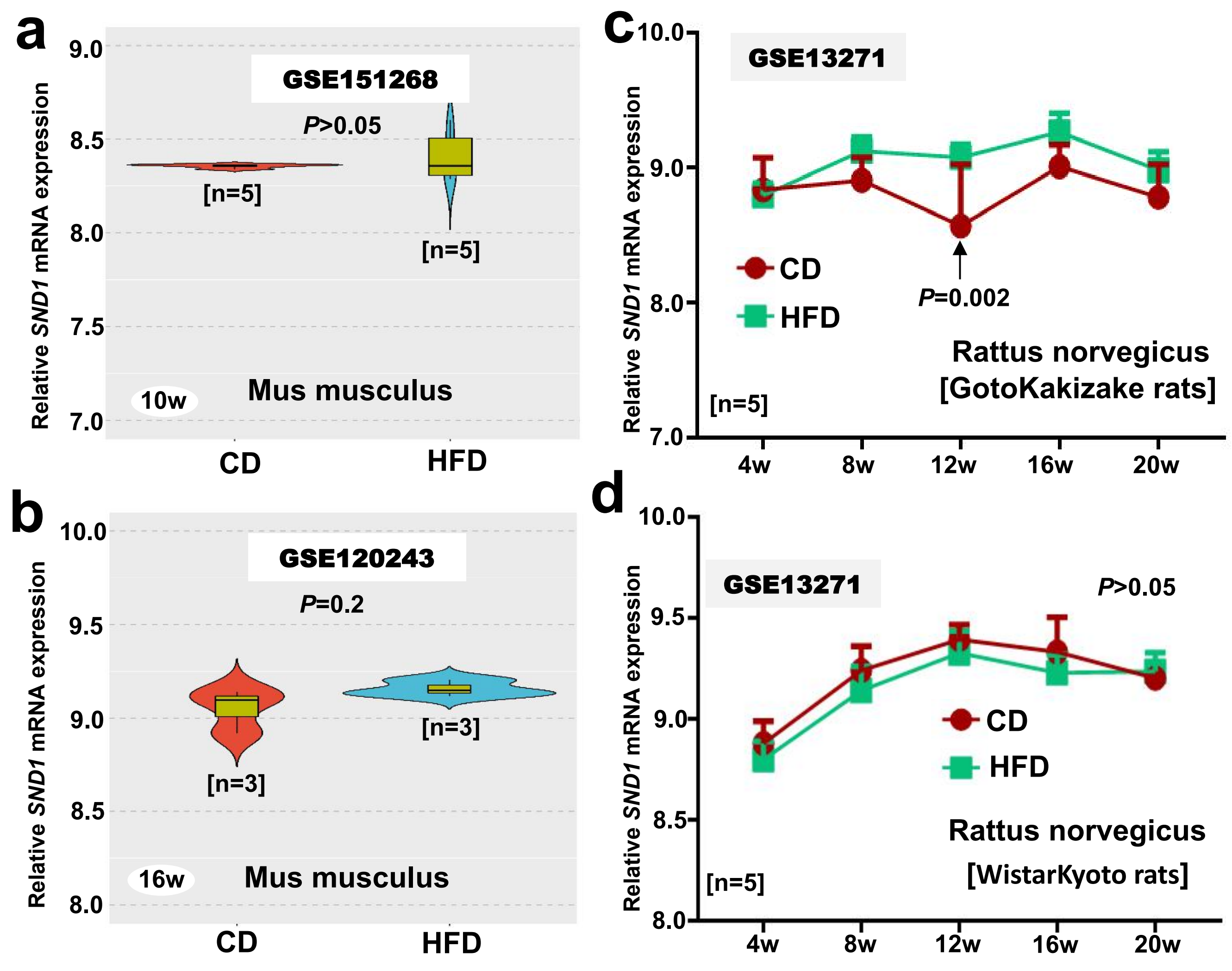

a
GSE151268
P>0.05
[n=5]
[n=5]
10w
Mus musculus
Relative SND1 mRNA expression
CD
HFD
b
GSE120243
P=0.2
[n=3]
[n=3]
16w
Mus musculus
Relative SND1 mRNA expression
CD
HFD
c
GSE13271
CD
HFD
P=0.002
Rattus norvegicus
[GotoKakizake rats]
[n=5]
Relative SND1 mRNA expression
4w
8w
12w
16w
20w
d
GSE13271
P>0.05
CD
HFD
Rattus norvegicus
[WistarKyoto rats]
[n=5]
Relative SND1 mRNA expression
4w
8w
12w
16w
20w

Figure S1

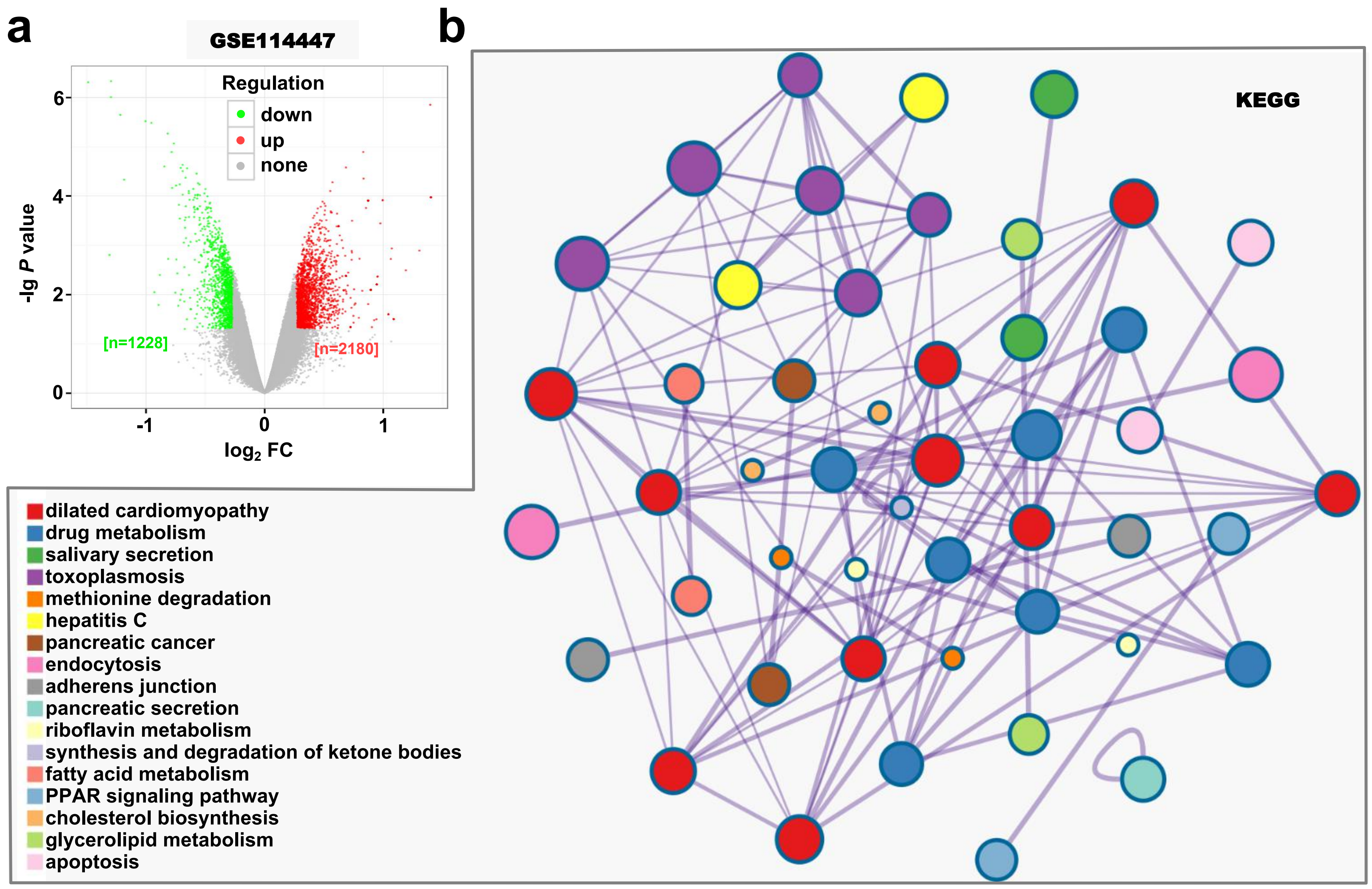
a
GSE114447
Regulation
down
up
none
-lg P value
[n=1228]
[n=2180]
log2 FC
b
KEGG
dilated cardiomyopathy
drug metabolism
salivary secretion
toxoplasmosis
methionine degradation
hepatitis C
pancreatic cancer
endocytosis
adherens junction
pancreatic secretion
riboflavin metabolism
synthesis and degradation of ketone bodies
fatty acid metabolism
PPAR signaling pathway
cholesterol biosynthesis
glycerolipid metabolism
apoptosis

Figure S2

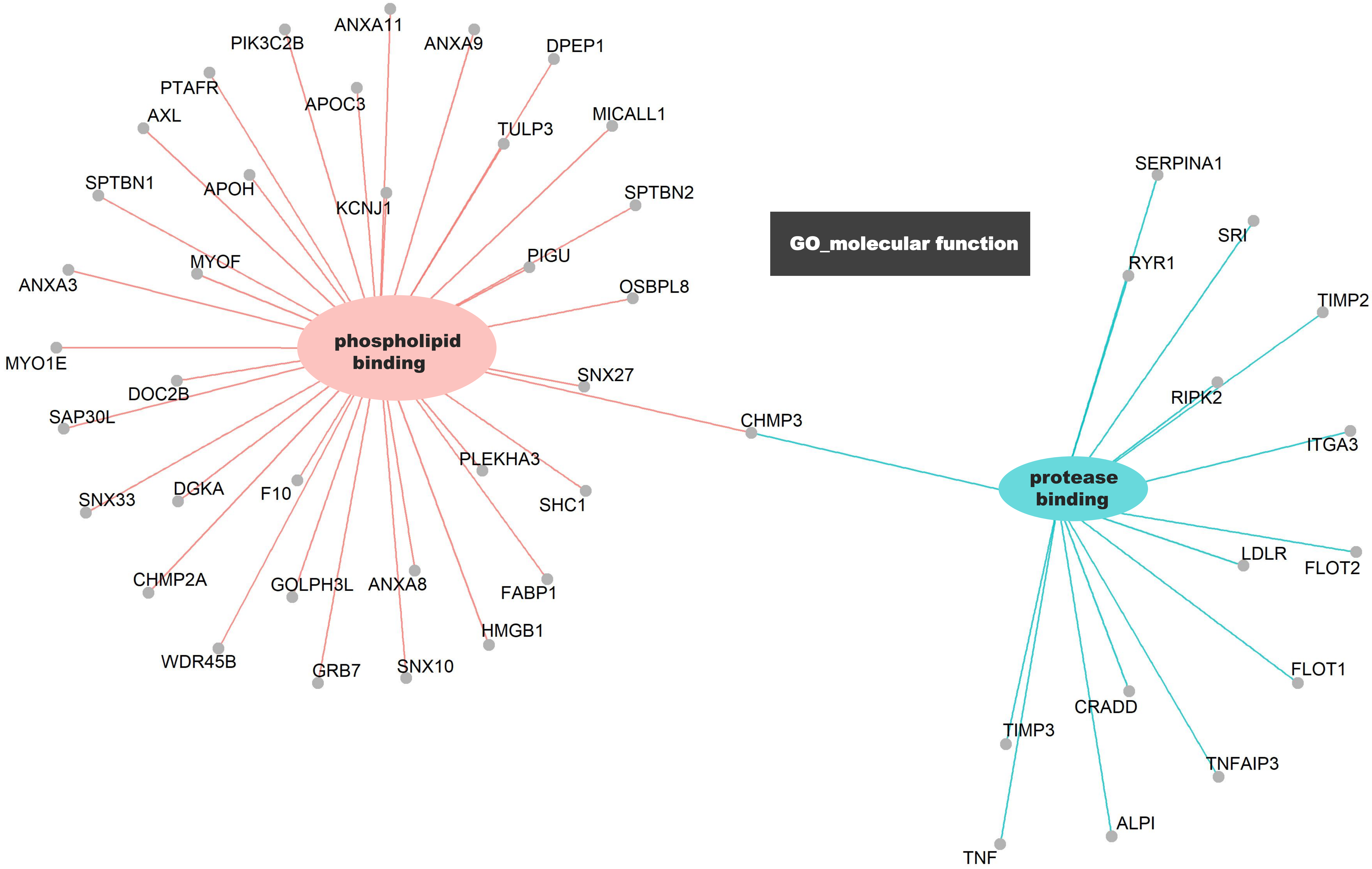

GO_molecular function
phospholipid binding
protease binding
ANXA11
PIK3C2B
ANXA9
DPEP1
PTAFR
APOC3
AXL
TULP3
MICALL1
SPTBN1
APOH
KCNJ1
SPTBN2
MYOF
PIGU
ANXA3
OSBPL8
MYO1E
SNX27
DOC2B
SAP30L
CHMP3
PLEKHA3
SNX33
DGKA
F10
SHC1
CHMP2A
GOLPH3L
ANXA8
FABP1
HMGB1
WDR45B
GRB7
SNX10
SERPINA1
SRI
RYR1
TIMP2
RIPK2
ITGA3
LDLR
FLOT2
FLOT1
CRADD
TIMP3
TNFAIP3
ALPI
TNF

Figure S3

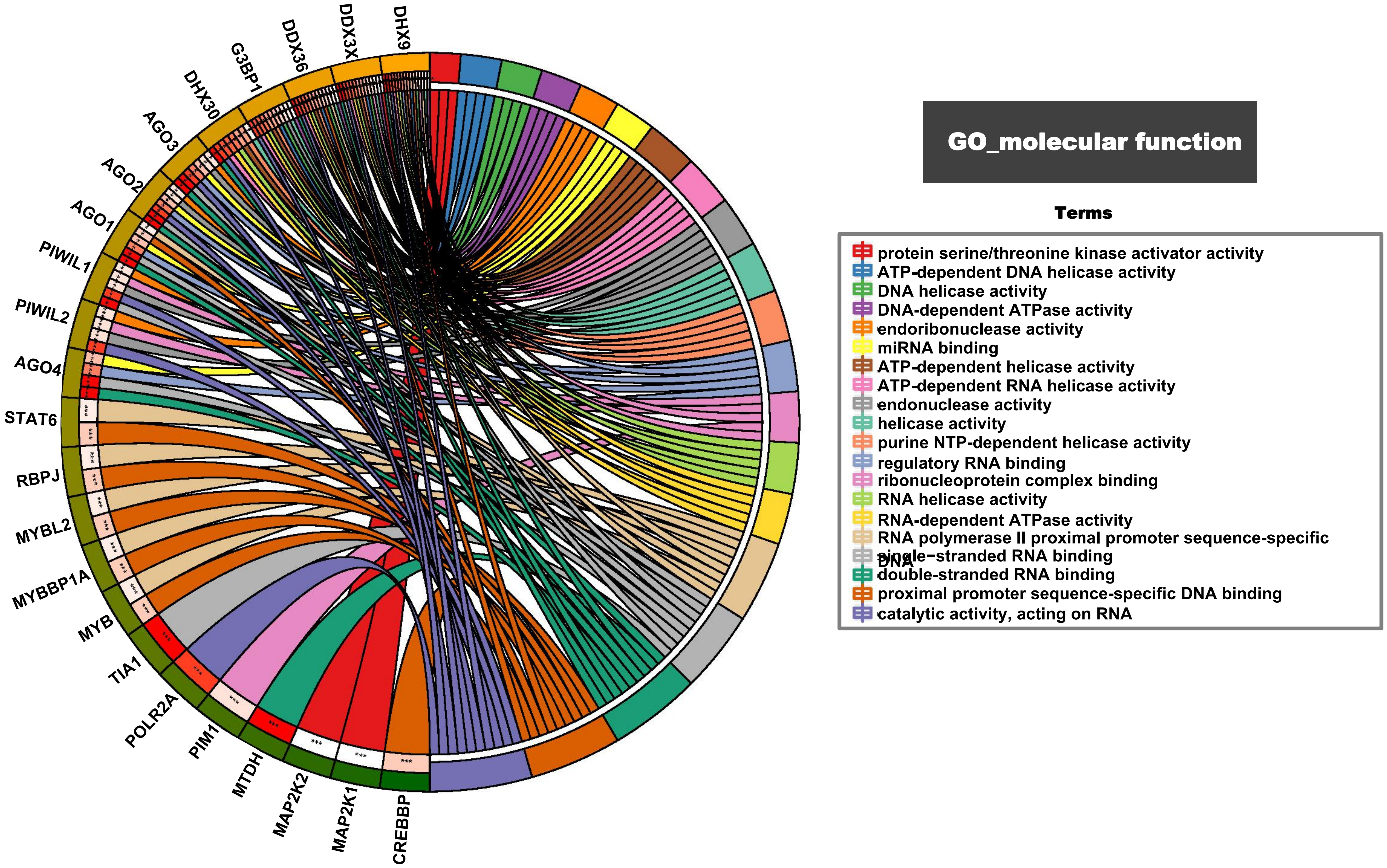

GO_molecular function
Terms
protein serine/threonine kinase activator activity
ATP-dependent DNA helicase activity
DNA helicase activity
DNA-dependent ATPase activity
endoribonuclease activity
miRNA binding
ATP-dependent helicase activity
ATP-dependent RNA helicase activity
endonuclease activity
helicase activity
purine NTP-dependent helicase activity
regulatory RNA binding
ribonucleoprotein complex binding
RNA helicase activity
RNA-dependent ATPase activity
RNA polymerase II proximal promoter sequence-specific DNA
single-stranded RNA binding
double-stranded RNA binding
proximal promoter sequence-specific DNA binding
catalytic activity, acting on RNA
DHX9
DDX3X
DDX36
G3BP1
DHX30
AGO3
AGO2
AGO1
PIWIL1
PIWIL2
AGO4
STAT6
RBPJ
MYBL2
MYBBP1A
MYB
TIA1
POLR2A
PIM1
MTDH
MAP2K2
MAP2K1
CREBBP

Figure S4

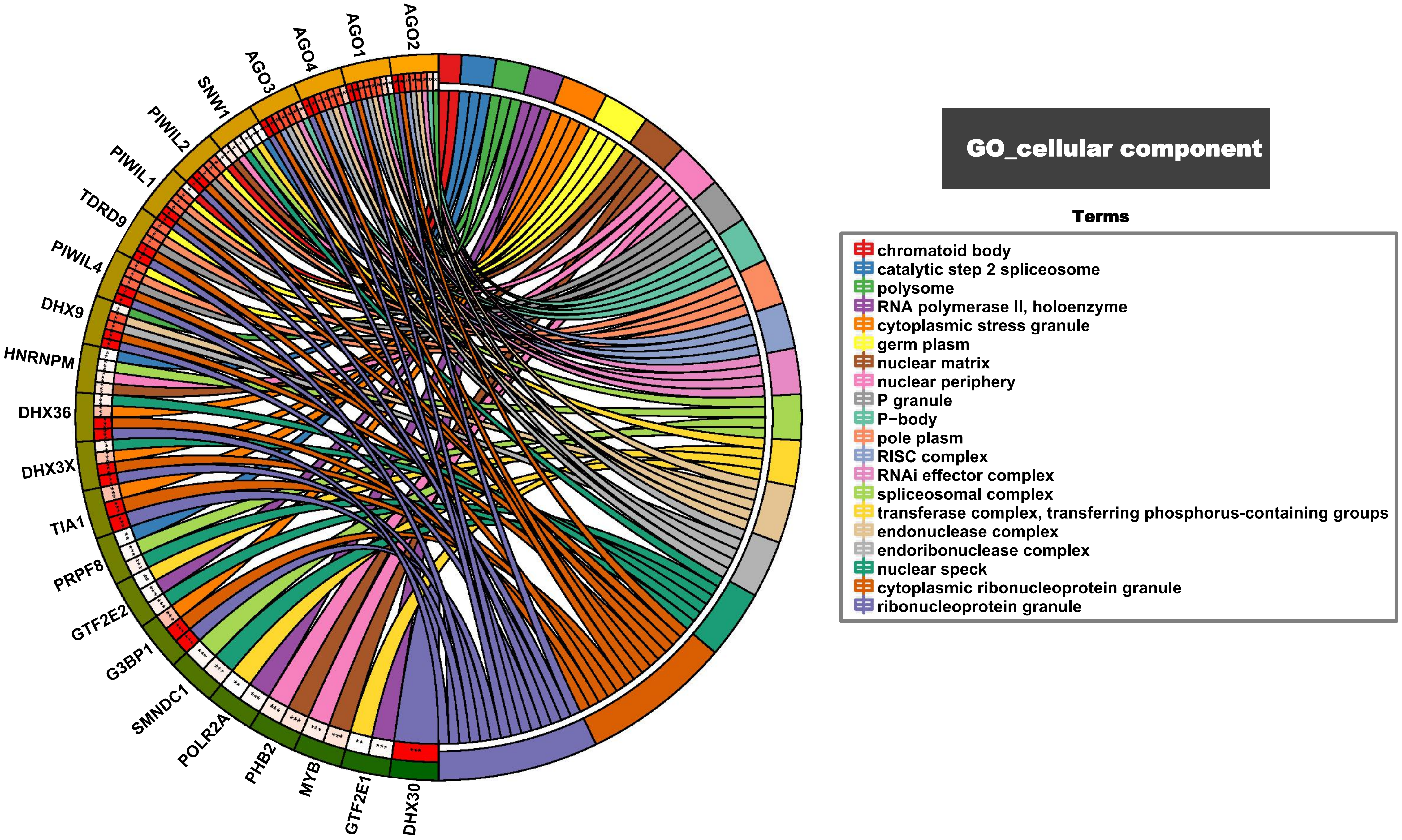
AGO2
AGO1
AGO4
AGO3
SNW1
PIWIL2
PIWIL1
TDRD9
PIWIL4
DHX9
HNRNPM
DHX36
DHX3X
TIA1
PRPF8
GTF2E2
G3BP1
SMNDC1
POLR2A
PHB2
MYB
GTF2E1
DHX30
GO_cellular component
Terms
chromatoid body
catalytic step 2 spliceosome
polysome
RNA polymerase II, holoenzyme
cytoplasmic stress granule
germ plasm
nuclear matrix
nuclear periphery
P granule
P−body
pole plasm
RISC complex
RNAi effector complex
spliceosomal complex
transferase complex, transferring phosphorus-containing groups
endonuclease complex
endoribonuclease complex
nuclear speck
cytoplasmic ribonucleoprotein granule
ribonucleoprotein granule

Figure S5

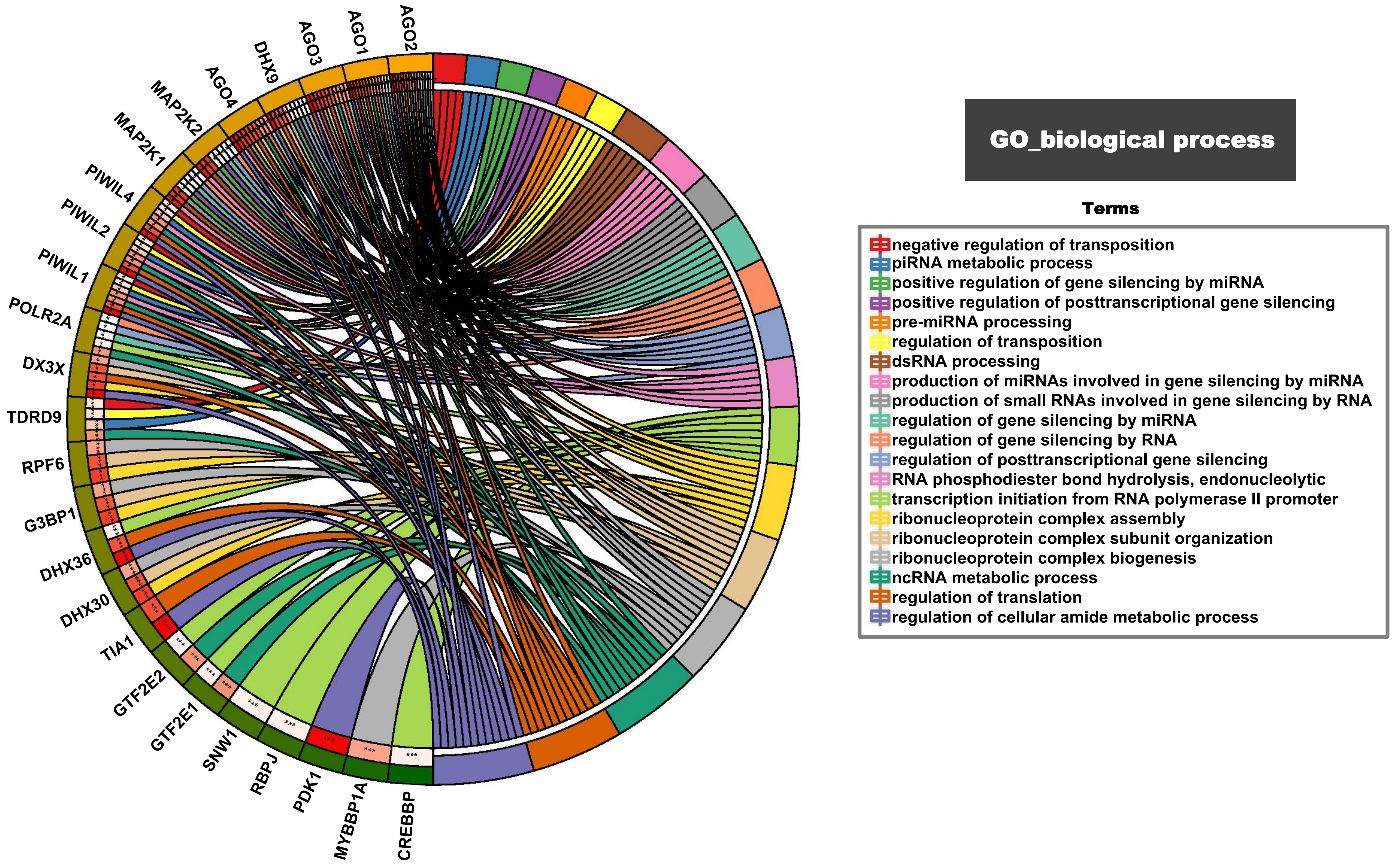

GO_biological process
Terms
negative regulation of transposition
piRNA metabolic process
positive regulation of gene silencing by miRNA
positive regulation of posttranscriptional gene silencing
pre-miRNA processing
regulation of transposition
dsRNA processing
production of miRNAs involved in gene silencing by miRNA
production of small RNAs involved in gene silencing by RNA
regulation of gene silencing by miRNA
regulation of gene silencing by RNA
regulation of posttranscriptional gene silencing
RNA phosphodiester bond hydrolysis, endonucleolytic
transcription initiation from RNA polymerase II promoter
ribonucleoprotein complex assembly
ribonucleoprotein complex subunit organization
ribonucleoprotein complex biogenesis
ncRNA metabolic process
regulation of translation
regulation of cellular amide metabolic process
AGO2
AGO1
AGO3
DHX9
AGO4
MAP2K2
MAP2K1
PIWIL4
PIWIL2
PIWIL1
POLR2A
DX3X
TDRD9
RPF6
G3BP1
DHX36
DHX30
TIA1
GTF2E2
GTF2E1
SNW1
RBPJ
PDK1
MYBBP1A
CREBBP

Figure S6

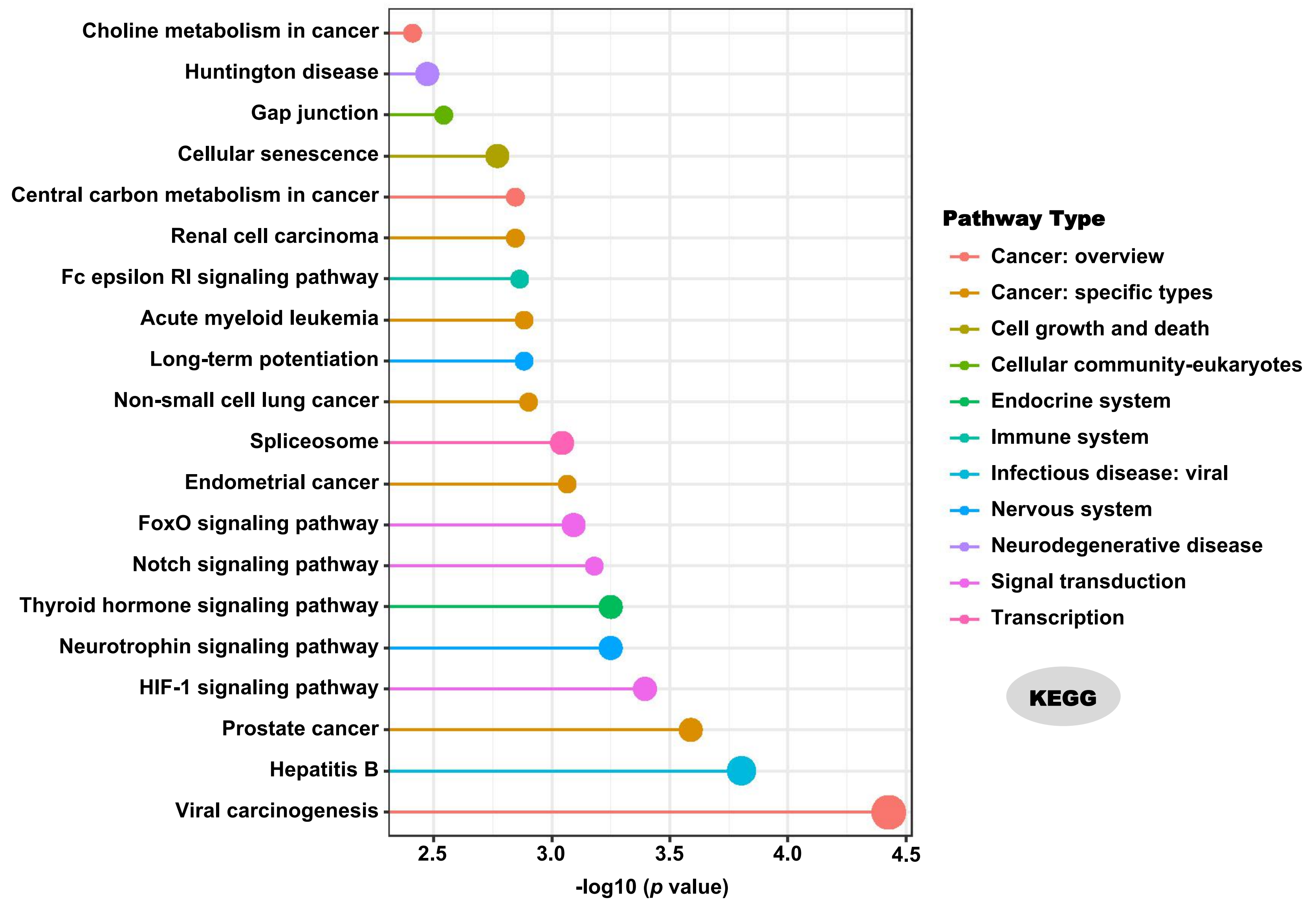
Choline metabolism in cancer
Huntington disease
Gap junction
Cellular senescence
Central carbon metabolism in cancer
Renal cell carcinoma
Fc epsilon RI signaling pathway
Acute myeloid leukemia
Long-term potentiation
Non-small cell lung cancer
Spliceosome
Endometrial cancer
FoxO signaling pathway
Notch signaling pathway
Thyroid hormone signaling pathway
Neurotrophin signaling pathway
HIF-1 signaling pathway
Prostate cancer
Hepatitis B
Viral carcinogenesis
2.5
3.0
3.5
4.0
4.5
-log10 (p value)
Pathway Type
Cancer: overview
Cancer: specific types
Cell growth and death
Cellular community-eukaryotes
Endocrine system
Immune system
Infectious disease: viral
Nervous system
Neurodegenerative disease
Signal transduction
Transcription
KEGG

Figure S7
